# Endometrial cancer-associated mutants of SPOP are defective in regulating estrogen receptor-*α* protein turnover

**DOI:** 10.1038/cddis.2015.47

**Published:** 2015-03-12

**Authors:** P Zhang, K Gao, X Jin, J Ma, J Peng, R Wumaier, Y Tang, Y Zhang, J An, Q Yan, Y Dong, H Huang, L Yu, C Wang

**Affiliations:** 1 State Key Laboratory of Genetic Engineering, Collaborative Innovation Center for Genetics and Development, School of Life Sciences, Fudan University, 220 Handan Road, Shanghai 200433, China; 2 Shanghai Cancer Center, Institutes of Biomedical Sciences, Fudan University, Shanghai 200032, China; 3 Department of Urology, Shanghai First People's Hospital, School of Medicine, Shanghai Jiaotong University, Shanghai 200080, China; 4 Department of Biochemistry and Molecular Biology, Mayo Clinic College of Medicine, Rochester, Minnesota 55905, USA; 5 Department of Obstetrics and Gynecology, Shanghai First People's Hospital, Shanghai Jiaotong University, Shanghai 200080, China; 6 Department of Pathology, The University of Arizona Medical Center, 1501N. Campbell Ave, Tucson, Arizona 85724, USA

## Abstract

Increasing amounts of evidence strongly suggests that dysregulation of ubiquitin-proteasome system is closely associated with cancer pathogenesis. Speckle-type POZ protein (SPOP) is an adapter protein of the CUL3-based E3 ubiquitin ligase complexes. It selectively recruits substrates for their ubiquitination and subsequent degradation. Recently, several exome-sequencing studies of endometrial cancer revealed high frequency somatic mutations in SPOP (5.7–10%). However, how SPOP mutations contribute to endometrial cancer remains unknown. Here, we identified estrogen receptor-*α* (ER*α*), a major endometrial cancer promoter, as a substrate for the SPOP-CUL3-RBX1 E3 ubiquitin ligase complex. SPOP specifically recognizes multiple Ser/Thr (S/T)-rich degrons located in the AF2 domain of ER*α*, and triggers ER*α* degradation via the ubiquitin-proteasome pathway. SPOP depletion by siRNAs promotes endometrial cells growth. Strikingly, endometrial cancer-associated mutants of SPOP are defective in regulating ER*α* degradation and ubiquitination. Furthermore, we found that SPOP participates in estrogen-induced ER*α* degradation and transactivation. Our study revealed novel molecular mechanisms underlying the regulation of ER*α* protein homeostasis in physiological and pathological conditions, and provided insights in understanding the relationship between SPOP mutations and the development of endometrial cancer.

Endometrial cancer is the most common gynecologic malignancy that arises from the endometrium, or lining, of the uterus. Endometrial cancer causes ~74 000 deaths annually among women worldwide.^1^ Most patients present with low-grade, early-stage disease. However, patients with more aggressive, high-grade tumors that spread beyond the uterus will usually progress within 1 year.^2^ For effective cancer prevention and treatment, it is necessary to identify genetic alterations that initiate endometrial cancer and contribute to its progression. Recently, significant progress has been made in identifying the genetic alterations in endometrial cancer using array-based technologies and next-generation sequencing.^3, 4, 5, 6^ Mapping the genomic landscape of endometrial cancer has produced comprehensive molecular classification of these tumors, which may ultimately serve to improve the diagnosis and treatment of patients with endometrial cancer.^7^ Among these investigations, speckle-type POZ protein (SPOP) was identified as one of the most frequently altered genes by somatic point mutations in endometrial cancers through large-scale exome-sequencing approaches.^3, 4, 5, 6^ Nonetheless, how SPOP mutations contribute to the pathogenesis and progression of endometrial cancer remains unknown.

SPOP is an adaptor protein of the CUL3-RBX1 E3 ubiquitin ligase complex. It selectively recruits substrates via its N-terminal MATH domain, whereas its BTB domain mediates dimerization and interaction with CUL3.^8^ SPOP has been linked to the ubiquitination of several substrates in both *Drosophila* and human cells, including the steroid receptor coactivator SRC-3, death domain-associated protein Daxx, the phosphatase Puc, the transcriptional regulator Ci/Gli, and several others.^9, 10, 11, 12, 13^ All endometrial cancer-associated SPOP mutations identified so far affect evolutionarily conserved residues in the MATH domain, suggesting that the mutations may alter the interaction of SPOP with its substrates.^3, 4, 5, 6^ In addition to endometrial cancer, SPOP is also mutated in 4.6 to 14.4% of patients with prostate cancer across different ethnic and demographic backgrounds.^14^ Importantly, mutual exclusivity of SPOP mutation with ETS family gene rearrangement, as well as a high association with CHD1 deletion reinforces SPOP mutation as defining a distinct molecular subclass of prostate cancer.^14, 15^

Estrogen receptor-*α* (ER*α*), encoded by *ESR1* gene, is a nuclear transcriptional factor that mediates estrogen-stimulated cell proliferation in hormone-responsive cancers, such as breast, endometrial and ovarian cancers.^16^ The ER*α* protein is highly overexpressed in breast, endometrial, and ovarian cancers and is among the first known targets for molecular therapy in any cancers.^16^ After binding to estrogen, ER*α* dimerizes and translocates into the nucleus, where it recruits co-activators or co-repressors, as well as chromatin-remodeling factors, to estrogen response elements on target gene promoters to activate or repress transcription.^17^ ER*α* is a member of the sex steroid receptors family that ligand-dependently regulates the functions of the sexual organs. Other sex steroid receptors include the androgen receptor (AR), the estrogen receptor*-β*, and the progesterone receptor. All sex steroid receptors are built with similar modular structure, including a DNA-binding domain, a hinge region with a nuclear location signal, and a ligand-binding domain.^18^ Recently, we reported that SPOP influences the AR stability and inhibits AR-mediated gene transcription and prostate cancer cell growth.^19^ Importantly, prostate cancer-associated mutants of SPOP cannot bind to AR and fail to promote AR ubiquitination and degradation, implying the importance of this pathway in the resistance to antiandrogen therapy of prostate cancer.^19^ On the basis of that SPOP regulates AR abundance, as well as similar domain architecture between ER*α* and AR, we investigated the possible role of SPOP in controlling ER*α* protein stability.

In this study, we demonstrated that SPOP forms a functional CUL3-SPOP-RBX1 E3 ubiquitin ligase complex which targets ER*α* for ubiquitination and proteasomal degradation in endometrial cancer cells. Moreover, this effect is abrogated by the endometrial cancer-associated SPOP mutations. Our results provide a functional insight into the molecular mechanism of endometrial cancer pathway involved with SPOP mutations.

## Results

### SPOP interacts with ER*α* in cells

**Figure 1:**
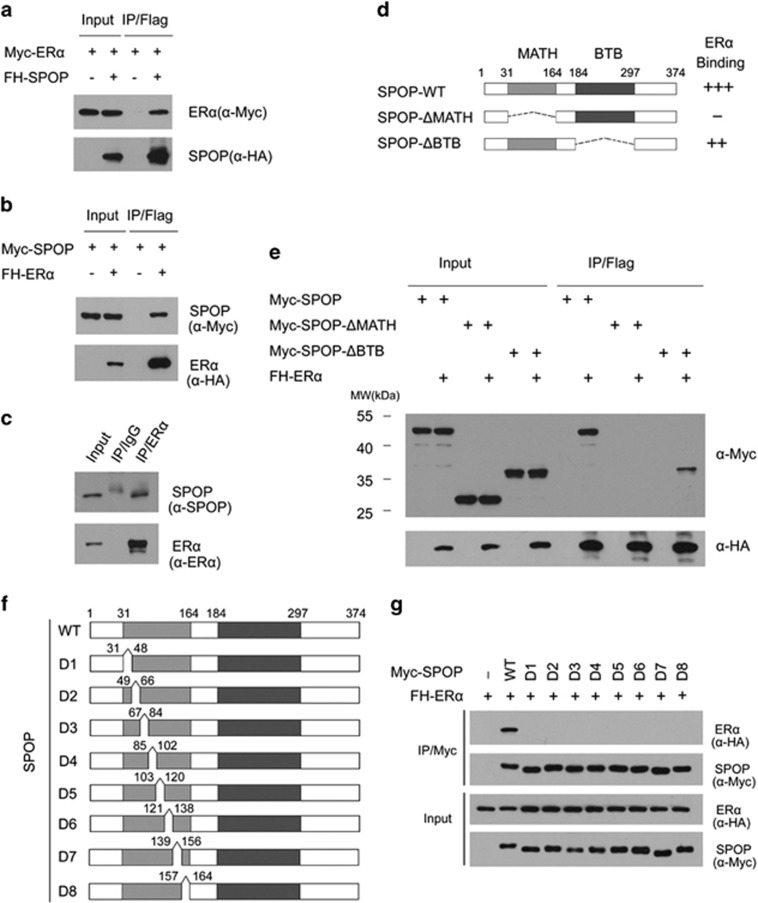
SPOP interacts with ER*α* in cells. (**a**,**b**) Ectopically expressed SPOP and ER*α* interact with each other. The 293T cells were co-transfected with FLAG-HA (FH)-SPOP and Myc-ER*α* constructs. After 24 h, cell lysates were prepared for co-IP with anti-FLAG antibody and WB analyzes. (**b**) co-IP assay was performed between ectopically expressed FH-ER*α* and Myc-SPOP. (**c**) Endogenous SPOP and ER*α* proteins interact with each other in Ishikawa cells. After being treated with 20 *μ*M MG132 for 4 h, cell lysates were prepared for co-IP with anti-ER*α* antibody and WB analyzes with indicated antibodies. (**d**) Schematic representation of SPOP deletion mutants. Binding capacity of SPOP to ER*α* is indicated with the symbol. (**e**) ER*α* binds to the MATH domain of SPOP. The 293T cells were co-transfected with FH-ER*α* and Myc-SPOP-WT or deletion mutants (ΔMATH, ΔBTB) constructs. After 24 h, cell lysates were prepared for co-IP assay with anti-FLAG antibody and WB analyzes. (**f**) Schematic representation of MATH domain deletion mutants of SPOP. Binding capacity of SPOP to ER*α* is indicated with the symbol. (**g**) The integrity MATH domain of SPOP is crtical for ER*α* binding. The 293T cells were co-transfected with FH-ER*α* and Myc-SPOP-WT or deletion mutants (D1–D8) constructs. After 24 h, cell lysates were prepared for co-IP assay with anti-Myc antibody and WB analyzes

It was previously reported that SPOP regulates AR stability.^19^ Because ER*α* is the most extensively studied biomarker and the best predictor for response to endocrine therapy in patients with endometrial cancer, we explored the possibility that SPOP regulates ER*α* protein stability via a similar mechanism as that of AR stability. Accordingly, we first examined whether SPOP interacts with ER*α* in cells. To do this, FLAG-HA (FH)-SPOP and Myc-ER*α* constructs were co-expressed in 293T cells. Cell lysates were subsequently prepared for co-immunoprecipitation (co-IP) with anti-FLAG antibody. As shown in Figure 1a, Myc-ER*α* was immunoprecipitated by FH-SPOP, suggesting an interaction between these two proteins. Similar results were also obtained in the reciprocal co-IP experiment in which FH-ER*α* was able to immunoprecipiate Myc-SPOP (Figure 1b). Next, we decided to extend our analysis by investigating whether endogenous SPOP and ER*α* can interact with each other. In this case, we chose Ishikawa cells, an ER*α*-positive human endometrial adenocarcinoma cell line, for subsequent study. Sanger sequencing of SPOP exon6/7 of Ishikawa cells revealed no mutation in MATH domain (data not shown). Immunoprecipitation using anti-ER*α* antibody was performed using cell lysates prepared from Ishikawa cells. As shown in Figure 1c, endogenous SPOP was efficiently immunoprecipitated by ER*α*, suggesting these two proteins can also interact with each other at endogenous levels.

SPOP contains two structural domains: a substrate-binding MATH domain at the N-terminus and a CUL3-binding BTB domain at the C-terminus.^8^ To determine which domain may mediate its interaction with ER*α*, we generated two deletion mutants of SPOP (SPOP-ΔBTB and ΔMATH), corresponding to the deletion of these two domains, respectively (Figure 1d). co-IP assay was performed to test the binding affinity of the full length SPOP (SPOP-WT) and the two deletion mutants with overexpressed ER*α* in 293T cells. As shown in Figure 1e, while SPOP-WT and SPOP-ΔBTB interacted efficiently with ER*α*, the interaction between SPOP-ΔMATH and ER*α* was totally abolished. To further map the ER*α*-binding sites in the MATH domain of SPOP, we constructed a series of small deletion mutants of SPOP in MATH domain (SPOP-D1–D8), then we compared the binding affinity of SPOP-WT and mutants (D1–D8) with ER*α*. As shown in Figures 1f and g, all SPOP mutants (D1–D8) completely lost ER*α* binding capacity, suggesting that the integrity of MATH domain was critical for binding to ER*α* efficiently. Therefore, this result suggests that the MATH domain is responsible for the interaction between SPOP and ER*α*. Taken together, our findings demonstrate that SPOP binds ER*α* in cells through the MATH domain.

### ER*α* is a *bona fide* substrate of the SPOP-CUL3-RBX1 E3 ubiquitin ligase complex

**Figure 2:**
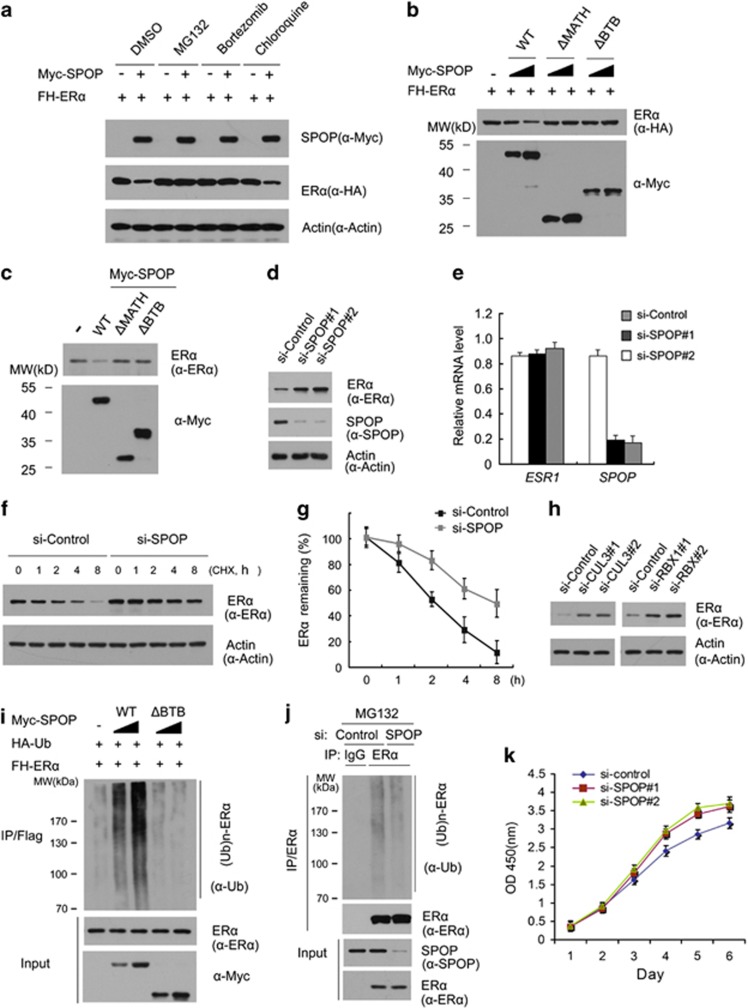
The SPOP-CUL3-RBX1 ubiquitin ligase complex targets ER*α* for ubiquitination and degradation. (**a**) SPOP regulates ER*α* protein levels through the proteasome pathway. The 293T cells were transfected with FH-ER*α* in combination with or without the Myc-SPOP constructs. After 24 h, cells were treated with MG132 (20 *μ*M), Bortezomib (200 nM), chloroquine (100 mM), or DMSO for 4 h before cell lysates were prepared for WB analyzes. Actin, a loading control. (**b**) The BTB and MATH domains in SPOP are essential for SPOP-mediated degradation of ER*α*. FH-ER*α* and different amounts of Myc-SPOP-WT or deletion mutants (ΔMATH, ΔBTB) constructs were transfected into 293T cells. After 24 h, cell lysates were prepared for WB analyzes. (**c**) SPOP regulates endogenous ER*α* protein levels. Ishikawa cells were transfected with Myc-SPOP-WT, or deletion mutants (ΔMATH, ΔBTB) constructs. After 24 h, cell lysates were prepared for WB analyzes. (**d**) Knockdown of SPOP increases endogenous ER*α* protein levels. Ishikawa cells were transfected with control or two SPOP-specific siRNAs. After 48 h, cell lysates were prepared for WB analyzes. (**e**) Quantitative RT-PCR measurement of the mRNA levels of *SPOP* and *ESR1* in SPOP-knockdown Ishikawa cells. The mRNA level of *GAPDH* was used for normalization. The mean values (S.D.) of three independent experiments are shown. (**f**,**g**) Knockdown of SPOP prolongs ER*α* protein half-life. Ishikawa cells were transfected with control or SPOP-specific siRNA. After 48 h, cells were chased with 30 *μ*M cycloheximide (CHX). At the indicated time points, cell lysates were prepared for WB analyzes. (**f**) At each time point, the intensity of ER*α* was first normalized to the intensity of Actin (loading control) and then to the value of the 0-h time point (**g**). The mean values (S.D.) of three independent experiments are shown. (**h**) Knockdown of RBX1 or CUL3 increases endogenous ER*α* protein levels. Ishikawa cells were transfected with control siRNA or siRNAs for RBX1 or CUL3. After 48 h, cell lysates were prepared for WB analyzes. (**i**) SPOP promotes ER*α* polyubiquitination *in vivo*. FH-ER*α*, HA-Ub, and Myc-SPOP-WT or ΔBTB mutant constructs were co-transfected into 293T cells. After 24 h, cells were treated with 20 *μ*M MG132 for 4 h. ER*α* proteins were immunoprecipitated with anti-FLAG antibody and resolved by SDS/PAGE. The ubiquitinated forms of ER*α* were analyzed by WB with anti-Ub antibody. (**j**) Knockdown of SPOP decreases ubiquitination of endogenous ER*α*. Ishiwaka cells were transfected control or SPOP-specific siRNA. After 48 h, cells were treated with 20 *μ*M MG132 for 4 h and then the same procedure was performed as **i**. (**k**) Knockdown of SPOP promotes Ishikawa cells growth. Ishikawa cells were transfected with control or two SPOP-specific siRNAs. After 48 h, the cell growth was measured by CCK-8 assay at indicated days. The mean values (S.D.) of three independent experiments are shown

Then we explored whether the SPOP-CUL3-RBX1 E3 ubiquitin ligase complex can promote the ubiquitination and degradation of ER*α*. As shown in Figure 2a, expression of SPOP decreased the ectopically co-expressed ER*α* protein level in 293T cells in a dose-dependent manner. This effect was completely blocked when cells were treated with the proteasome inhibitors MG132 or Bortezomib (Figure 2a). In contrast, lysosome inhibitor chloroquine could not block SPOP-mediated ER*α* degradation. These results indicated that SPOP downregulates ER*α* protein via the proteasomal and not the lysosomal degradation pathway. Moreover, SPOP-WT, but not the SPOP-ΔBTB or SPOP-ΔMATH mutant, promoted ER*α* degradation (Figure 2b), indicating that the BTB and MATH domains are both required for SPOP-mediated ER*α* degradation. Next, we examined the effect of SPOP on the degradation of endogenous ER*α*. Similarly, as shown in Figure 2c, overexpression of SPOP-WT, but not the SPOP-ΔBTB or SPOP-ΔMATH mutant in Ishikawa cells resulted in a moderate decrease in the protein level of endogenous ER*α*. Moreover, knockdown of endogenous SPOP using two gene-specific siRNAs increased ER*α* protein levels in Ishikawa cells (Figure 2d). To exclude the possibility that ER*α* protein elevation resulted from transcriptional upregulation, we performed qRT-PCR to measure the mRNA levels of SPOP and ER*α* in SPOP-depleted Ishikawa cells. In contrast to the significant decrease of SPOP mRNA levels, the mRNA levels of *ESR1* gene (encoding ER*α*) in SPOP-depleted Ishikawa cells stayed at a level similar to that of the control cells (Figure 2e), indicating that the effect of SPOP on ER*α* protein levels is not mediated through the upregulation of ER*α* mRNA levels. In addition, knockdown of SPOP remarkably prolonged the half-life of endogenous ER*α* protein in Ishikawa cells (Figures 2f and g), further suggesting that SPOP regulates ER*α* protein at the post-translational level.

Next we sought to determine whether other subunits of the SPOP-CUL3-RBX1 E3 ubiquitin ligase complex are also required for ER*α* degradation. We respectively knocked down RBX1 or CUL3 using two gene-specific siRNAs and examined the changes in ER*α* protein level in Ishikawa cells. As shown in Figure 2h, knockdown of either RBX1 or CUL3 resulted in a significant increase in ER*α* protein levels, an effect similar to SPOP knockdown. These results suggest that other subunits of ubiquitin ligase complex are also required for the degradation of ER*α*.

To further determine whether ER*α* was degraded through SPOP-mediated polyubiquitination, HA-Ubiquitin and FH-ER*α* constructs were co-expressed in 293T cells with different doses of SPOP-WT or SPOP-ΔBTB mutant. As shown in Figure 2i, ER*α* protein was robustly polyubiquitinated by the co-expressed SPOP-WT in a dose-dependent manner. In contrast, little or no ER*α* polyubiquitination was observed in SPOP-ΔBTB expressing cells (Figure 2i). Accordingly, knockdown of endogenous SPOP in Ishikawa cells decreased the polyubiquitination of endogenous ER*α* (Figure 2j). ER*α* is a key pro-oncogenic transcriptional factor in endometrial cancer. It activates target genes that promote cell proliferation or decrease apoptosis. As SPOP regulates ER*α* protein abundance in Ishikawa cells, we measured the cells growth of SPOP-depleted Ishikawa cells. As shown in Figure 2k, knockdown of SPOP by two gene-specific siRNAs promote Ishikawa cells growth when compared with control knockdown cells. Similar effects were observed in other two endometrial cell lines, RL95-2 (ER*α* positive), and KLE cells (ER*α* negative; Supplementary Figures S1A, 1B). Sanger sequencing of SPOP exon6/7 of RL95-2 and KLE cells revealed no mutation in MATH domain (data not shown). The results that SPOP knockdown promote the cells growth of ER*α* negative endometrial cells suggested SPOP may regulate other oncoproteins in endometrial cells in addition to ER*α*.

Taken together, these data demonstrate that the SPOP-CUL3-RBX1 E3 ubiquitin ligase complex regulates ER*α* stability through ubiquitin-dependent proteasomal degradation pathway in endometrial cancer cell lines. It also suggested SPOP may regulate endometrial cells growth in an ER*α*-dependent and independent manner.

### Multiple Ser/Thr (S/T)-rich motifs in ER*α* are required for SPOP-mediated ER*α* degradation

**Figure 3:**
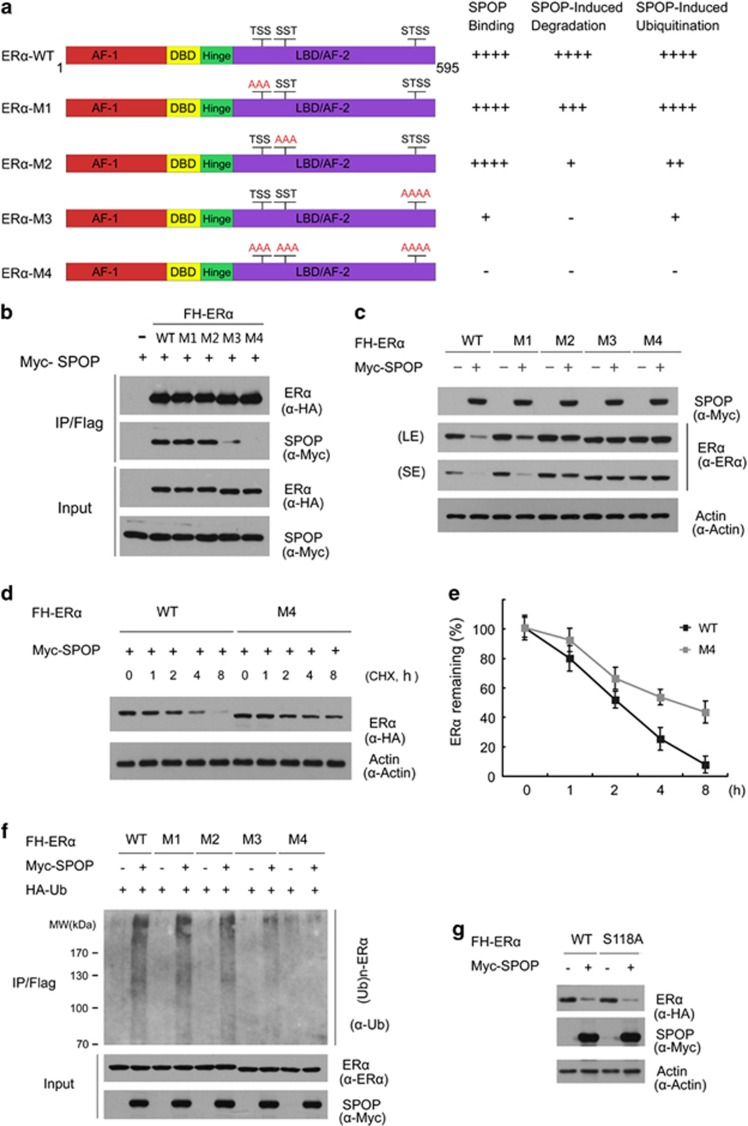
The S/T-rich motifs in ER*α* are degrons recognized by SPOP. (**a**) Schematic representation of wild-type ER*α* protein with the upper contiguous Ser/Thr residues indicating the S/T-rich motifs in its amino-acid sequence. The ER*α* point mutants (M1, M2, M3, and M4) were constructed starting from the FH-ER*α*-WT vector are schematically reported below the wild-type protein. On the right of each schematic protein is summarized its SPOP-binding capacity, sensitivity to SPOP-induced degradation or ubiquitination. (**b**) The S/T-rich motifs in ER*α* are required for its binding to SPOP. The 293T cells were transfected with the indicated constructs. After 24 h, cell lysates were prepared for co-IP assay with anti-FLAG antibody and WB analyzes. (**c**) The S/T-rich motifs in ER*α* are required for SPOP-mediated ER*α* degradation. The 293T cells were transfected with the indicated constructs. After 24 h, cell lysates were prepared for WB analyzes. SE, short exposure; LE, long exposure. (**d**, **e**) Mutation of the S/T-rich motifs prolongs the half-life of ER*α*. ER*α*-WT or M4 mutant was transfected into 293T cells. After 24 h, cells were treated with 30 *μ*M CHX. At the indicated time points, cell lysates were prepared for WB analyzes (**d**). At each time point, the intensity of ER*α* was first normalized to the intensity of Actin and then to the value of the 0-h time point (**e**). (**f** )The S/T-rich motifs are required for SPOP-mediated ER*α* polyubiquitination. The 293T cells were transfected with the indicated constructs. After 24 h, cells were treated with 20 *μ*M MG132 for 4 h and the cell lysates were prepared for co-IP assay with anti-FLAG antibody and WB analyzes. The mean values (S.D.) of three independent experiments are shown. (**g**) Ser118 of ER*α* is not required for SPOP-mediated ER*α* degradation. The 293T cells were transfected with FH-ER*α* or S118A mutant in combination with or without Myc-SPOP constructs. After 24 h, cell lysates were prepared for WB analyzes

Previous study has reported that an optimal SPOP-binding motif contains 3–4 contiguous Ser/Thr (S/T) residues.^11^ Similar S/T-rich motifs are present in known SPOP-binding proteins, such as Puc, MacroH2A, Ci/Gli, SRC-3, and AR. Therefore, we examined the protein sequence of ER*α* and found three S/T-rich motifs located in the ligand-binding domain/AF2 domain resembling the SPOP-binding pattern (Figure 3a). In order to examine whether these S/T-rich motifs are actually required for SPOP-ER*α* interaction, we generated three ER*α* mutants (M1, M2, and M3) in which the Ser/Thr residues in each motif were mutated to Ala (A). An ER*α*-M4 mutant was also generated in which all 10 Ser/Thr residues in 3 motifs were mutated to Ala (A; Figure 3a). The 293T cells were co-transfected with SPOP and wild-type ER*α* or one of these mutants. Co-IP assays demonstrated that SPOP was co-immunoprecipitated by ER*α*-WT, M1, or M2 mutant at similar levels, whereas the interaction was significantly reduced between SPOP and ER*α*-M3 mutant. Moreover, the interaction was completely abolished between SPOP and ER*α*-M4 mutant (Figure 3b). Thus, these results suggested that the S/T-rich motifs of ER*α* were essential for SPOP binding. Each S/T-rich motif may partially contribute to the SPOP-binding capacity, but the third S/T-rich motif appeared most critical for the ER*α*-SPOP interaction.

Next, we examined whether the S/T-rich motifs are required for SPOP-mediated ER*α* degradation. As shown in Figure 3c, mutation of the first S/T-rich motif (ER*α*-M1) mildly reduced SPOP-mediated ER*α* degradation when compared with ER*α*-WT, whereas mutation of the second S/T-rich motif (ER*α*-M2) severely reduced SPOP-mediated ER*α* degradation. In addition, mutation of the third or all S/T-rich motifs (ER*α*-M3 or M4) completely abrogated SPOP-mediated ER*α* degradation (Figure 3c). These results suggested that the S/T-rich motifs are important in SPOP-mediated ER*α* degradation. To further explore whether the S/T-rich motifs are also important for ER*α* turnover, we measured the half-lives of the ER*α*-WT or ER*α*-M4 mutant in 293T cells. As shown in Figures 3d and e, ER*α*-M4 mutant exhibited a significantly prolonged half-life than that of ER*α*-WT. To further determine the importance of these motifs as degrons, ER*α*-WT or mutants was co-transfected with or without SPOP in 293T cells. *In vivo* ubiquitination assay demonstrated that mutation of all S/T-rich motifs (ER*α*-M4) rather than any single motif (ER*α*-M1, M2, or M3) totally abolished SPOP-mediated ER*α* ubiquitination (Figure 3f).

ER*α* ubiquitination can be mediated by multiple E3 ubiquitin ligases in response to changing cellular conditions.^16^ E6-AP is the most extensively studied E3 ligase for ER*α*, and its loss attenuates 17*β*-estradiol (E2)-induced ER*α* degradation.^20^ Previous studies reported E6-AP binding to ER*α* in a Ser118-dependent manner.^20^ However, our results showed that SPOP could efficiently target ER*α*-S118A for degradation to the same extent as ER*α*-WT (Figure 3g), suggesting the Ser118 is dispensable for SPOP-mediated ER*α* degradation. Taken together, these observations demonstrate that the three S/T-rich motifs functions as ER*α* degrons, which are essential for SPOP binding and subsequent degradation of ER*α*, and the third S/T-rich motif is most crucial for this function.

### Endometrial cancer-associated mutants of SPOP are defective in promoting ER*α* degradation and ubiquitination

**Figure 4:**
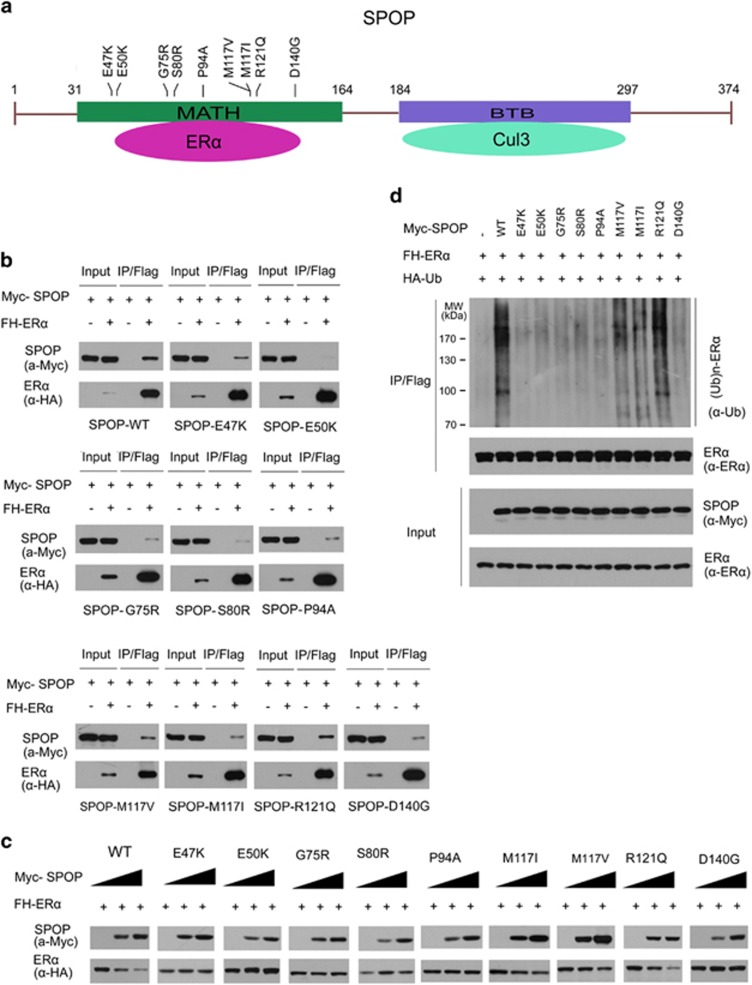
Endometrial cancer-associated mutants of SPOP are defective in promoting ER*α* degradation and ubiquitination. (**a**) Distribution of the point mutations on the SPOP gene found in endometrial cancer samples. These mutations are exclusively located in the N-terminal MATH domain of SPOP. (**b**) Endometrial cancer-associated mutants of SPOP are defective in promoting ER*α* degradation. 293T cells were transfected with FH-ER*α* and wild-type or mutated SPOP constructs as indicated. After 24 h, cell lysates were prepared for WB analyzes. (**c**) Endometrial cancer-associated mutants of SPOP are defective in interacting with ER*α*. The 293T cells were transfected with the indicated constructs. After 24 h, cell lysates were prepared for co-IP assay with anti-FLAG antibody and WB analyzes. (**d**) Endometrial cancer-associated mutants of SPOP are defective in promoting ER*α* ubiquitination. The 293T cells were transfected with the indicated constructs. After 24 h, cells were treated with 20 *μ*M MG132 for 4 h and cell lysates were prepared for immunoprecipitation and WB analyzes

SPOP was mutated in 5.7–10% of patients with endometrial cancer across multiple independent cohorts (Table 1). Moreover, SPOP mutations were detected in three major histological subtypes of endometrial cancer (endometrioid, clear cell and serous). Notably, all the SPOP mutations found in endometrial cancer far-exclusively occur in the MATH domain which is responsible for ER*α* binding (Figure 4a). Therefore, we propose that endometrial cancer-associated SPOP mutations may cause dysfunction in regulating ER*α* protein level. To test this, nine Myc-tagged endometrial cancer-associated mutants of SPOP were generated according to four large-scale exome-sequencing studies (Table 1), including E47K, E50K, G75R, S80R, P94A, M117V, M117I, R121Q, and D140G. We initially examined their interactions with ER*α* by co-IP assay. As shown in Figure 4b, mutations of the residues at MATH domain substantially decreased the capacity of SPOP to interact with ER*α in vivo,* suggesting the endometrial cancer-associated SPOP mutants are defective in binding to ER*α*. We then examined the capacities of endometrial cancer-associated SPOP mutants in promoting ER*α* degradation. As shown in Figure 4c, a group of SPOP mutants (SPOP-M117V, M117I, and R121Q) displayed reduced capacity to promote ER*α* degradation when compared with SPOP-WT, whereas other groups did not alter ER*α* protein level (SPOP-E47K, E50K, G75R, P94A, and D140G) or increased ER*α* protein level (SPOP-S80R), suggesting that endometrial cancer-associated SPOP mutants may differentially regulate ER*α* stability. Furthermore, *in vivo* ubiquitination assays indicated that some SPOP mutants (SPOP-E47K, E50K, G75R, S80R, and D140G) lost the capacity to promote ER*α* polyubiquitination, whereas SPOP-M117V, M117I, and R121Q mutants could generate partially polyubiquitinated form of ER*α* (Figure 4d). Taken together, our findings suggest that endometrial cancer-associated mutants of SPOP are defective in promoting ER*α* degradation and ubiquitination.

We then explored the impact of SPOP mutations on endometrial cell proliferation. First, we established Ishikawa cell lines that were stably transfected with control constructs, SPOP-WT or SPOP mutants, respectively. SPOP expression levels were determined by WB analyzes. As shown in Supplementary Figure S2A, FH-SPOP-WT or mutants were stably expressed in Ishikawa cells. We found stable overexpression of SPOP-WT in Ishikawa cells resulted in a marked decrease in the protein level of endogenous ER*α*, whereas stable overexpression of SPOP mutants did not alter the endogenous ER*α* protein level (Supplementary Figure S2A). Next, we measured the cells growth of Ishikawa stable cell lines using CCK-8 assay. As shown in Supplementary Figure S1B, stable overexpression of SPOP-WT, but not the SPOP mutants, resulted in a retardation of Ishikawa cell growth. Interestingly, a subset of SPOP mutants (S80R, M117V, M117I, and R121Q, D140G) accelerated Ishikawa cells growth when compared with control cells, suggesting a possible gain-of-function dominant-negative effect of these SPOP mutants on the Ishikawa cells growth (Supplementary Figure S2B). Thus, our findings suggest that wild-type SPOP can suppress endometrial cell proliferation, and this suppression is abrogated by endometrial cancer-associated SPOP mutations.

### SPOP participated in estrogen-induced ER*α* degradation

**Figure 5:**
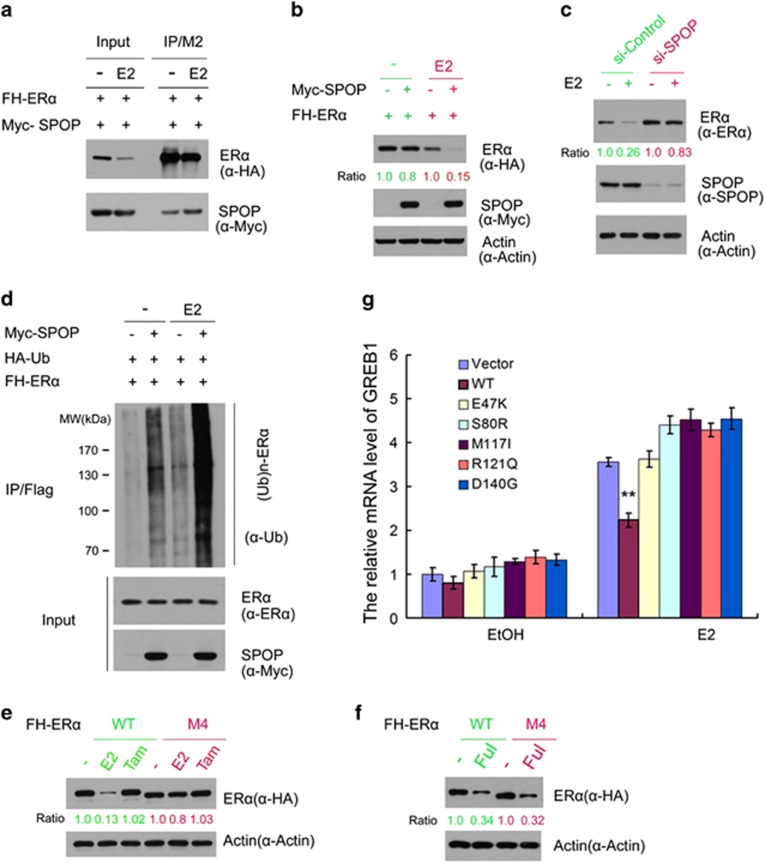
Estrogen potentiates SPOP-mediated degradation of ER*α*. (**a**) Estrogen enhances the SPOP-ER*α* interaction. FH-ER*α* and Myc-SPOP constructs were co-transfected into 293T cells. After 24 h, cells were treated with the vehicle ethanol (EtOH,−) or 10 nM 17*β*-estradiol (E2) for 4 h before cell lysates were prepared for co-IP and WB analyzes. (**b**) Estrogen enhances SPOP-mediated ER*α* degradation. The 293T cells were transfected with the indicated constructs. A small amount of Myc-SPOP constructs was used in transfection. After 24 h, cells were treated with the vehicle ethanol (EtOH) or 10 nM 17*β*-estradiol (E2) for 4 h before cells lysates were prepared for WB analyzes. The density of ER*α* was determined by normalizing to actin (loading control) first and then to the normalized value in mock-treated cells. (**c**) Knockdown of SPOP attenuates estrogen-induced degradation of ER*α*. Ishikawa cells were transfected with control or SPOP-specific siRNA. After 48 h, cells were then treated with the vehicle ethanol (EtOH,−) or 10 nM 17*β*-estradiol (E2) for 4 h before cell lysates were prepared for WB analyzes. (**d**) Estrogen potentiates SPOP-induced polyubiquitination of ER*α*. The 293T cells were transfected with the indicated constructs. After 24 h, cells were treated with the vehicle ethanol (EtOH,−) or 10 nM 17*β*-estradiol (E2). Cells were then treated with MG132 for 4 h before cell lysates were prepared for IP and WB analyzes. (**e**) Ishikawa cells lines that stably transfected with control, SPOP-WT or SPOP mutants constructs were treated with 10 nM 17*β*-estradiol (E2) for 24 h. The mRNA level of ER*α* target gene *GREB1* was measured by qRT-PCR. The mRNA level of *GAPDH* was used for normalization. The mean values (S.D.) of three independent experiments are shown. **indicates statistical significance (**P<0.01). (**f**, **g**) Differential effects of estrogen on the protein level of ER*α*-WT and the SPOP degradation-resistant mutant (ER*α*-M4). The 293T cells were transfected with FH- ER*α*-WT or M4 mutant construct. After 24 h, cells were treated with vehicle ethanol (EtOH,−), 10 nM 17*β*-estradiol (E2), 10 nM Tamoxifen (Tam), and 10 nM Fulvestrant (Ful) for 4 h before cell lysates were prepared for WB analyzes

ER*α* is rapidly ubiquitinated and degraded through the ubiquitin-proteasome pathway after estrogen binding.^21^ However, the molecular mechanism by which estrogen downregulates ER*α* protein is not fully understood. To determine whether SPOP has a role in this process, we first examined whether estrogen treatment can affect SPOP-ER*α* interaction. FH-ER*α* and Myc-SPOP were overexpressed in 293T cells, and then the cells were treated with hormone 17*β*-estradiol (E2) or vehicle ethanol (EtOH) before harvesting. co-IP assay was performed to test the impact of estrogen treatment on SPOP-ER*α* interaction. As shown in Figure 5a, E2 treatment leads to a significant downregulation of overexpressed ER*α*. However, more SPOP was co-immunoprecipitated by ER*α*, suggesting that estrogen treatment enhances the interaction between ER*α* and SPOP. We further demonstrated that SPOP-induced downregulation of ER*α* protein was markedly enhanced by E2 treatment (Figure 5b). Furthermore, we demonstrated that knockdown of endogenous SPOP largely diminished E2-induced downregulation of endogenous ER*α* protein in Ishikawa cells (Figure 5c). Consistent with these findings, E2 treatment resulted an increase of SPOP-mediated ER*α* ubiquitination (Figure 5d). Finally, we demonstrated that stable overexpression of SPOP-WT but not the SPOP mutants diminished E2–dependent transactivation of ER*α* target gene *GREB1* in Ishikawa cells (Figure 5e). Similar effects were observed in other two ER*α* target genes, *Cyclin D1* and *ABCA3* (Supplementary Figure S3A and 3B). We also demonstrated that SPOP-WT and mutants differentially regulated the protein levels of GREB1 and Cyclin D1 (Supplementary Figure S3C).

We then examined whether the estrogenic effect on SPOP regulation of ER*α* degradation is mediated through the S/T-rich motifs. As shown in Figure 5f, E2 but not the Tamoxifen treatment significantly decreased the protein level of ER*α*-WT. However, this effect was largely diminished with the ER*α*-M4 mutant, which can't bind SPOP (Figure 5f). The anti-estrogen drug fulvestrant causes immobilization of ER*α* in the nuclear matrix accompanied by rapid degradation by the ubiquitin-proteasome pathway.^22, 23^ We also examined whether SPOP is involved in fulvestrant-induced ER*α* degradation. As shown in Figure 5g, ER*α*-M4 mutant remained susceptible to degradation induced by fulvestrant, suggesting fulvestrant-induced ER*α* Degradation occurs through SPOP-independent mechanisms. Taken together, these data suggest that SPOP has an important role in estrogen-induced ER*α* degradation and transactivation in endometrial cancer cells.

## Discussion

**Figure 6:**
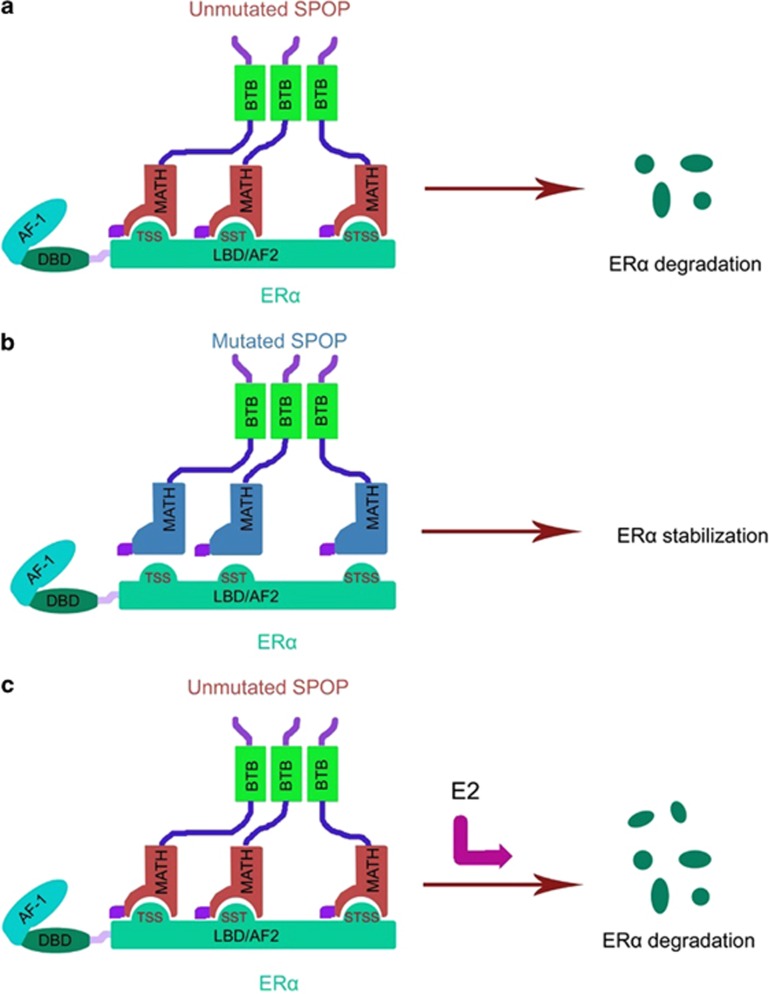
Models depicting SPOP-mediated ER*α* degradation in physiological and pathological conditions in endometrial cancer. (**a**) Unmutated SPOP promotes degradation of wild-type ER*α*. (**b**) Endometrial cancer-associated mutants of SPOP are defective in promoting ER*α* ubiquitination and degradation. (**c**) Estrogen potentiates SPOP-mediated ER*α* degradation

Recurrent SPOP mutations in endometrial cancer have been confirmed by four independent genome-wide studies (Table 1). Although frequent mutations of SPOP in endometrial cancer have been identified, the functional impact of these mutations remains unknown. In this study, we demonstrated that ER*α* is a *bona fide* substrate for the SPOP-CUL3-RBX1 E3 ubiquitin ligase complex (Figure 6). SPOP recognizes the Ser/Thr-rich degrons in the AF2 domain of ER*α*, and promotes ER*α* ubiquitination and proteasomal degradation. Endometrial cancer-associated mutants of SPOP are defective in promoting ER*α* degradation and ubiquitination. Moreover, SPOP participates in estrogen-induced ER*α* degradation. Taken together, these findings provide new insights in our understanding of the physiological and pathophysiological significance of SPOP in regard to the development of endometrial cancer.

Mining of the cancer exome-sequencing data deposited in COSMIC (Catalouge of Somatic Mutations in Cancer) database (http://www.sanger.ac.uk/cosmic) revealed that SPOP mutations are common in endometrial and prostate cancers, but rare in cancers of other tissue types. The copy number analyzes of amplification, loss of heterozygosity (LOH), and deletion in 24 cancer types revealed LOH at high percentages in the SPOP locus, suggesting genomic loss of the SPOP locus occurs frequently in human cancers.^9^ Thus, multiple mechanisms such as somatic mutations, LOH, and epigenetic silencing might be utilized by different cancer types to inactivate SPOP. Another unexpected finding is that SPOP mutational spectra are entirely different between endometrial and prostate cancer. The endometrial cancer-associated SPOP mutants were not observed in previous prostate cancer data. Reciprocally, prostate cancer-associated SPOP mutants were not observed in endometrial cancer data. An in-depth understanding of this difference is still lacking. A plausible explanation is that the SPOP mutants from different cancers might show differential effect on the regulation of certain substrates. Further investigation is needed to determine which substrates and related signaling pathways were dysregulated in SPOP mutated endometrial or prostate cancer.

Through detailed mutation analysis, we identified three S/T-rich motifs located in the AF2 domain of ER*α*. Our results suggested that SPOP-mediated degradation and ubiquitination of ER*α* may depend on cooperatively among multiple S/T-rich motifs. Notably, multiple S/T-rich degrons are also found in several other SPOP substrates, such as Puc and Ci. For example, Puc contains one optimal and two suboptimal degrons.^24^ The results of *in vitro* ubiquitination with Puc mutants indicated that the relative contributions of S/T-rich motifs of Puc are proportional to their abilities to bind SPOP.^24^ A similar situation was observed for SPOP-ER*α* interaction (Figures 3b and f). Moreover, recent studies reported that SPOP can form large oligomers.^25^ SPOP oligomerization can serve to enhance SPOP-substrate avidity through the presentation of multiple substrate-binding MATH domains. Reciprocally, SPOP-substrate avidity would further enhanced by multiple S/T-rich motifs within a single substrate, a property especially common among the various SPOP substrates.^25^ So it is possible that oligomeric SPOP engages multiple S/T-rich motifs of ER*α* for ubiquitination and degradation.

Another important finding of our study is that estrogen induces ER*α* degradation by facilitating SPOP-ER*α* interaction. Although the detailed molecular basis for this remains unknown, one possible explanation is that, when estrogen bind to ER*α*, ER*α* undergoes conformation changes, thereby affecting the binding of the S/T-rich motifs to SPOP and subsequent ER*α* degradation. Interestingly, we found that ER*α*-M3 mutant protein showed a retarded electrophoretic motility compared with ER*α*-WT, whereas such a mobility shift was not observed in ER*α*-M1 or M2 mutant (Figure 3c), suggesting the third S/T-rich motif might be important for ER*α* conformation. This result is consistent with the finding that only ER*α*-M3 mutant is totally resistant to SPOP-mediated ER*α* degradation (Figure 3c). Our studies also demonstrated that SPOP knockdown largely diminished but not abolished estrogen-induced ER*α* degradation (Figure 5c). Previous studies reported that E6-AP knockdown also attenuates estrogen-induced ER*α* degradation,^20^ suggesting multiple E3 ubiquitin ligases may participate in this process. Unlike estrogen, the anti-estrogen drug fulvestrant induces a distinct conformational change in the ER*α* and inhibits receptor homo-dimerization, nuclear localization, and enhances rapid degradation of the ER*α*.^26^ This process is driven by a SPOP-independent manner because ER*α*-M4 mutant protein that cannot bind SPOP remained susceptible to fulvestrant-induced degradation (Figure 5f).

For future studies, it will be useful to generate mice models of conditional endometrial-specific SPOP knockout or knockin to further characterize the phenotype of SPOP mutations *in vivo*, and determine whether ER*α* pathway is dysregulated in SPOP mutated endometrial cancer.

## Materials and Methods

### Cell culture, treatments, and transfection

Ishikawa, RL95-2, KLE and 293T cells were obtained from the American Type Culture Collection (ATCC, Manassas, VA, USA). The 293T cells were maintained in DMEM medium with 10% FBS, whereas Ishikawa, RL95-2, and KLE cells were maintained in DMEM/F12 medium with 10% FBS. Cells were transiently transfected using Lipofectamine RNAiMAX (for siRNA transfection) or 3000 (for plasmids transfection) (Invitrogen, Carlsbad, CA, USA) according to the manufacturer's instructions. In experiments involving treatment with 17*β*-estradiol (E2), Tamoxifen, fulvestrant, or vehicle ethanol (EtOH), cells were placed in phenol-red-free medium with 10% dextran-coated, charcoal-stripped FBS for 48 h prior to treatment with hormone or vehicle.

### Expression constructs

The HA-SPOP vectors were kindly provided by Dr. Masatoshi Hagiwara (Tokyo Medical and Dental University, Japan) and subcloned into pCIN4-FLAG-HA and pCMV-Myc expression vectors. The ER*α* cDNA was purchased from Genechem (Shanghai, China), and subcloned into pCIN4-FLAG-HA and pCMV-Myc expression vectors. ER*α* mutants and SPOP mutants were generated by QuickChange site-directed mutagenesis kit (Stratagene, La Jolla, CA, USA).

### RNA interference

Non-specific control siRNA and siRNAs for human SPOP, CUL3, and RBX1 were purchased from GenePharma (Shanghai, China). siRNA transfection of cells was performed following the manufacturer's instructions. The siRNA oligos sequences for SPOP are: si-SPOP1#1: 5′-GGAUGAUGUAAAUGAGCAA-3′. si-SPOP#2: 5′- GGACAGCGACTCTGAATCT-3′. The sequence of negative control is: si-Control: 5′-ACAGACUUCGGAGUACCUG-3′.

### Antibodies

The following antibodies were used: SPOP (ab137537; Abcam, Cambridge, UK), GREB1 (ab72999; Abcam), Cyclin D1 (ab134175; Abcam), ER*α* (8644; Cell Signaling, Beverly, MA, USA), ubiquitin (6652-1; Epitomics, Burlingame, CA, USA), Myc (9E10; Sigma Aldrich, St. Louis, MO, USA), FLAG (M2; Sigma), actin (AC-74; Sigma) and HA (MM5-101R; Millipore, Darmstadt, Germany).

### SPOP exon6/7 sequencing

The sanger sequencing strategy to detect SPOP exon6/7 sequence was performed as previously described.^14^ Genomic DNA from cell samples was extracted using the QIAamp DNA Mini Kit (QIAGEN GmbH, Hilden, Germany) following the manufacturer's instruction. PCR amplification and sequence of exon6/7 of SPOP was performed with the primers listed in supplementary Table 1.

### Immunoprecipitation

To immunoprecipitate the ectopically expressed FLAG-tagged proteins, transfected cells were lysed 24 h post-transfection in BC100 buffer. The whole-cell lysates were immunoprecipitated with the monoclonal anti-FLAG antibody-conjugated M2 agarose beads (Sigma) at 4 °C overnight. After three washes with FLAG lysis buffer, followed by two washes with BC100 buffer, the bound proteins were eluted using FLAG-Peptide (Sigma)/BC100 for 3 h at 4 °C. The eluted material was resolved by SDS-PAGE. To immunoprecipitate the endogenous proteins, cells were lysed with 1 × cell lysis buffer (Cell Signaling), and the lysate was centrifuged. The supernatant was precleared with protein A/G beads (Sigma) and incubated with indicated antibody overnight. Thereafter, protein A/G beads were applied, all at 4 °C. After 2 h of incubation, pellets were washed five times with lysis buffer and resuspended in sample buffer and analyzed by SDS-PAGE.

### Western blot

Cell lysates or immunoprecipitates were subjected to SDS-PAGE and proteins were transferred to nitrocellulose membranes (GE Healthcare, Little Chalfont, UK). The membranes were blocked in Tris-buffered saline (pH 7.4) containing 5% non-fat milk and 0.1% Tween-20, washed twice in TBS containing 0.1% Tween-20, and incubated with primary antibody for 2 h and followed by secondary antibody for 1 h at room temperature. The proteins of interest were visualized using ECL chemiluminescence system (Santa Cruz, Santa Cruz, CA, USA).

### Quantitative RT-PCR

Total RNA was isolated from transiently transfected cells using the TRIzol reagent (Tiangen, Shanghai, China), and cDNA was reversed-transcribed using the Superscript RT kit (TOYOBO, Osaka, Japan), according to the manufacturer's instructions. PCR amplification was performed using the SYBR Green PCR master mix Kit (TOYOBO). All quantization were normalized to the level of endogenous control GAPDH.

### Cell proliferation assay

Cell proliferation rate was determined using Cell Counting Kit-8 (CCK-8) according to the manufacturer's protocol (Dojindo Laboratories, Kumamoto, Japan). Briefly, The Ishikawa cells were seeded onto 96-well plates at a density of 2000 cells per well. During a 2–7 day culture periods, 10 *μ*l of the CCK-8 solution was added to cell culture and incubated for 2 h. The resulting color was assayed at 450 nm using a microplate absorbance reader (Bio-Rad, Hercules, CA, USA). Each assay was carried out in triplicate.

## Figures and Tables

**Table 1:** The somatic mutations of SPOP identified in exome sequencing of endometrial cancers

	**Amino-acid change**	**Mutation types**	**Histological subtypes**	**ratios**	**Reference**
1	G75R	Missense	Serous	10% (1/10)	Kuhn E *et al.*^6^
2	M117V D140G	Missense	Serous	5.9% (2/34)	Zhao S *et al.*^5^
3	E47K S80R P94A M117I R121Q	Missense	Serous Clear cell	8% (4/52) 9% (2/23)	Le Gallo M *et al.*^7^
4	E50K M117I R121Q D140G	Missense	Endometrioid Serous	5.7% (10/175) 7% (3/43)	Kandoth C *et al.*^4^

## Abbreviations

ER*α*: estrogen receptor-*α*
SPOP: Speckle-type POZ protein
Co-IP: co-immunoprecipitation
WB: Western blot
E2: 17*β*-estradiol
AR: androgen Receptor
EtOH: ethanol
