# Supplementary material for: Endometrial cancer-associated mutants of SPOP are defective in regulating estrogen receptor-α protein turnover
Source: Cell Death Dis. 2015 Mar 12;6(3):e1687–. doi: 10.1038/cddis.2015.47 (PMC4385925; doi:10.1038/cddis.2015.47)
Supplement: Supplementary Information [file cddis201547x1.doc]

**Table S1. Primers used for real-time RT-PCR, SPOP or ESR1 construction, SPOP exon 6/7 sequencing.**

| **SYBR®Green Quantitative RT-PCR primers** | |
| --- | --- |
| ESR1 | F: 5'-GGCTACATCATCTCGGTTCC -3'  R: 5'- TCAGGGTGCTGGACAGAAA -3' |
| GREB1 | F: 5’-CCAAGAATAACCTGTTGGCCCTGC -3’  R: 5’-GACATGCCTGCGCTCTCATACTTA -3’ |
| ABCA3 | F : 5'- TTCTTCACCTACATCCCCTAC -3'  R : 5'- CCTTTCGCCTCAAATTTCCC -3' |
| ESR1 | F: 5'-GGCTACATCATCTCGGTTCC -3'  R: 5'- TCAGGGTGCTGGACAGAAA -3' |
| SPOP | F : 5'- AGCAAATGATAAACTGAAAT -3'  R : 5'- GTCATCAGGGAGAAGCCCGT -3' |
| **SPOP mutation construction Primers** | |
| SPOP-ΔBTB(31-164) | F: 5'- CAAGATTCTGTCAACATTTCTGGCCAG -3'  R:5'- CACTACCTTGATCTGTGTGTAGCACCAAC -3' |
| SPOP-ΔMATH(184-297) | F: 5'- TCCGTGGAGAACGCTGCAGAAATTCTC -3'  R:5'- - CTCAGGAACCTTTACCATGTTCATGG 3' |
| SPOP-M1 Δ (31-48) | F: 5'- GGTGAAGTCATTAAAAGTTCTACATTTTCATC -3'  R:5'- CACTACCTTGATCTGTGTGTAGCACC -3' |
| SPOP-M2 Δ (49-66) | F: 5'- TGGTGTTTGCGAGTAAACCCCAAAGGG -3'  R:5'- CATTTCCTCCCGGCAAAAGCTAAAGTTATTGATG -3' |
| SPOP-M3 Δ (67-84) | F: 5'- TCACTTTACCTGTTACTGGTCAGCTGTC -3'  R:5'- TTTCAGTTTATCATTTGCTCCTGATGAAAATGTAG -3' |
| SPOP-M4 Δ (85-102) | F: 5'- AAATTCTCCATCCTGAATGCCAAGGGAG -3'  R:5'- CAGGTAATCTTTGCTTTCTTCATCTAAC -3' |
| SPOP-M5 Δ (103-120) | F: 5'- CGGGCATATAGGTTTGTGCAAGGCAAAGAC -3'  R:5'- GAATTTTGCCCGAACTTCACTCTTTGGAC -3' |
| SPOP-M6 Δ (121-138) | F: 5'- AGAGATTTTCTTTTGGATGAGGCCAACG -3'  R:5'- TTGACTCTCCATAGCTTTGGTTTCTTCTC -3' |
| SPOP-M7 Δ (139-156) | F: 5'- CTCTTCTGCGAGGTGAGTGTTGTGCAAG -3'  R:5'- ACGGATGAATTTCTTGAATCCCCAGTC 3' |
| SPOP-M8 Δ (157-164) | F: 5'- CAAGATTCTGTCAACATTTCTGGCCAG -3'  R:5'- GGTAAGCTTGTCATCAGGGAGAAGC -3' |
| SPOP-E47K | F: 5'- AAATGGGTGAAGTCATTAAAAGTTCTAC -3'  R:5'- TCTCCCGGCAAAAGCTAAAGTTATTGATGGTC -3' |
| SPOP-E50K | F: 5'- AAAGTCATTAAAAGTTCTACATTTTCATCAGGAGC -3'  R:5'- ACCCATTTCCTCCCGGCAAAAGCTAAAG -3' |
| SPOP-G75R | F: 5'- CGGTTAGATGAAGAAAGCAAAGATTACCTGTC -3'  R:5'- TTTGGGGTTTACTCGCAAACACCATTTCAG -3' |
| SPOP-S80R | F: 5'- GAAAGATTACCTGTCACTTTACCTGTTAC -3'  R:5'- CTTTCTTCATCTAACCCTTTGGGGTTTACTC -3' |
| SPOP-P94A | F: 5'- CAAAGAGTGAAGTTCGGGCAAAATTCAAATTCTC -3'  R:5'- CACAGCTGACCAGTAACAGGTAAAGTGAC -3' |
| SPOP-M117I | F: 5'- AGAGAGTCAACGGGCATATAGGTTTGTGC -3'  R:5'- ATAGCTTTGGTTTCTTCTCCCTTGGC -3' |
| SPOP-M117V | F: 5'- GTGGAGAGTCAACGGGCATATAGGTTTGTG -3'  R:5'- AGCTTTGGTTTCTTCTCCCTTGGCATTC -3' |
| SPOP-R121Q | F: 5'- AGGCATATAGGTTTGTGCAAGGCAAAGAC -3'  R:5'- GTTGACTCTCCATAGCTTTGGTTTCTTCTC -3' |
| SPOP-D140G | F: 5'- GTTTTCTTTTGGATGAGGCCAACGGGCTTCTC -3'  R:5'- CTCTACGGATGAATTTCTTGAATCCCCAGTC -3' |
| **ESR1 mutation construction Primers** | |
| ESR1-M1 | F: 5'- CAGCTCGGTTCCGCATGATGAATCTG -3'  R:5'- CTGCAGCCAGCAGCATGTCGAAGATCTC -3' |
| ESR1-M2 | F: 5'- GCCGCCCTGAAGTCTCTGGAAGAGAAGGAC -3'  R:5'- GGCCAGAAATGTGTACACTCCAGAATTAAGC -3' |
| ESR1-M3 | F: 5'- CTGCAGCGCATTCCTTGCAAAAGTATTACATCACG -3'  R:5'- CAGCGCCCGCAGTGGCCAAGTGGCTTTGGTCCGTC -3' |
| ESR1-S118A | F: 5'- GCGCCTTTCCTGCAGCCCCACGGCCAGC -3'  R:5'- CAGCTGCGGCGGCGGGTGCAGTAGCATC -3' |
| **SPOP mutation detection Primers** | |
| SPOP-Exon6 (Amp) | F: 5'- ACCCATAGCTTTGGT TTCTTCTCCC -3'  R: 5'- TATCTG TTTTGG ACAGGTGTTTGCG -3' |
| SPOP-Exon6 (Seq) | R:5'- TATCTGTTTTGG ACAGGTGTTTGCG -3' |
| SPOP-Exon7(Amp) | F: 5'- ACTCATCAGATCTGGGAA CTGC -3'  R: 5'- AGTTGTGGCTTTGATCTGGTT -3' |
| SPOP-Exon7(Seq) | F:5'- ACTCATCAGATCTGGGAACTGC -3' |

**Figure S1. Knockdown of SPOP promotes RL95-2 and KLE cells growth.** RL95-2 (A) and KLE cells (B) were transfected with control or two SPOP-specific siRNAs. After 48 hr, the cells growth was measured by CCK8 assay at indicated days.

**Figure S2. SPOP-WT and mutants differentially regulate Ishikawa cells proliferation.** (A) WB analyses for FH-SPOP-WT or mutants in stably transfected Ishikawa cells. SE, short exposure; LE, long exposure. (B) The proliferation of Ishikawa cells stably transfected with control, SPOP-WT or SPOP mutant constructs were measured by CCK8 assay at indicated days.

**Figure S3. SPOP-WT and mutants differentially regulate the expression of ERα target genes.** (A, B) Ishikawa cells lines that stably transfected with control, SPOP-WT or SPOP mutants (SPOP-E47K, S80R, M117I, R121Q and D140G) constructs were treated with the vehicle ethanol (EtOH,-) or 10 nM 17β-estradiol (E2) for 24 hr. The mRNA level of ERα target gene *Cyclin D1* (A) and *ABCA3* (B) were measured by qRT-PCR. The mRNA level of GAPDH was used for normalization. The mean values (S.D.) of three independent experiments are shown. * indicates statistical significance (*, p < 0.01). (C) Similar as (A, B), the protein levels of GREB1 and Cyclin D1 were analyzed by WB with indicated antibodies.
