# Supplementary figures and images for: Endometrial cancer-associated mutants of SPOP are defective in regulating estrogen receptor-α protein turnover
Source: Cell Death Dis. 2015 Mar 12;6(3):e1687–. doi: 10.1038/cddis.2015.47 (PMC4385925; doi:10.1038/cddis.2015.47)

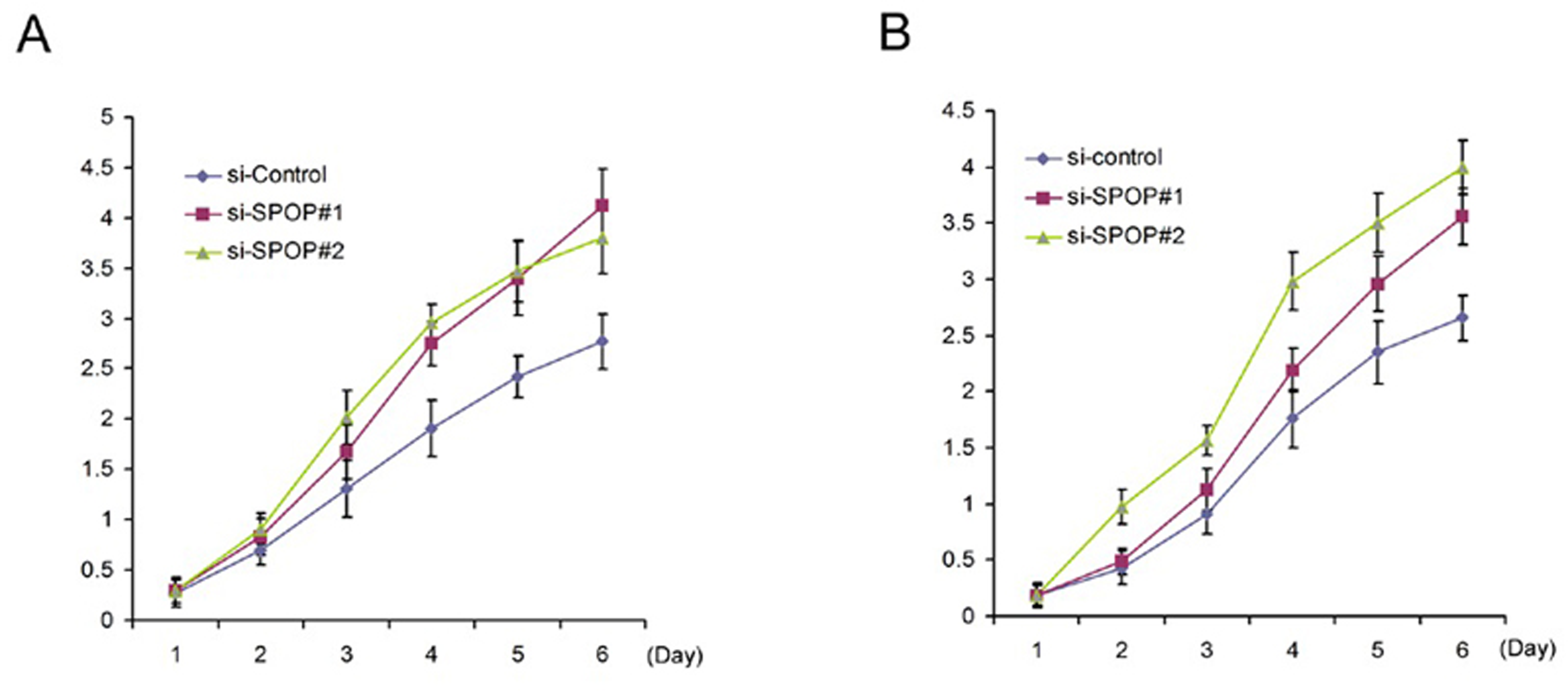

Supplement: Supplementary Figure 1 [file cddis201547x2.tif]

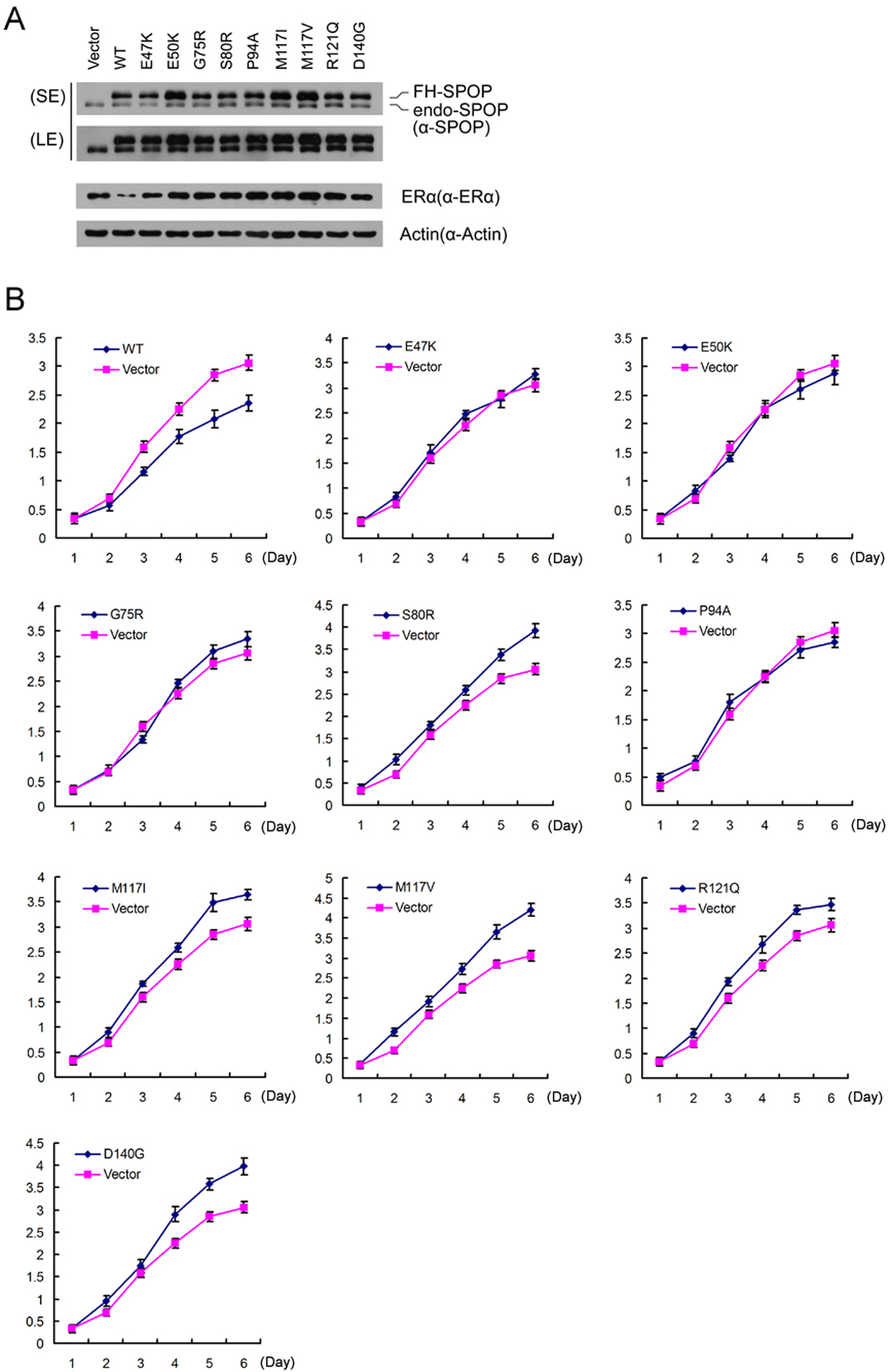

Supplement: Supplementary Figure 2 [file cddis201547x3.tif]

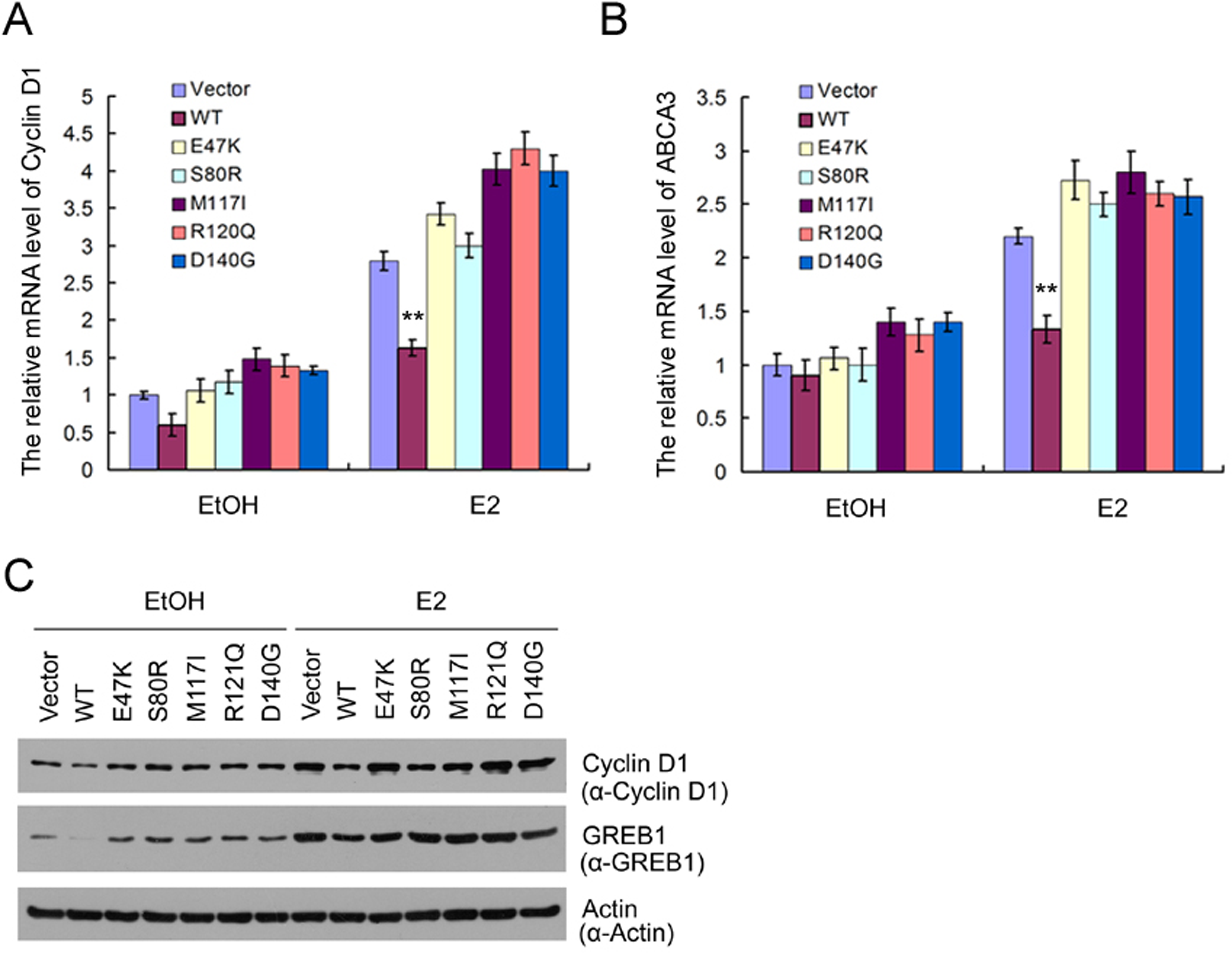

Supplement: Supplementary Figure 3 [file cddis201547x4.tif]
